# Assessing the automaticity of moral processing: Efficient coding of moral information during narrative comprehension

**DOI:** 10.1080/17470210802254441

**Published:** 2008-08-08

**Authors:** Fionnuala C. Murphy, Gemma Wilde, Neil Ogden, Philip J. Barnard, Andrew J. Calder

**Affiliations:** Medical Research Council Cognition and Brain Sciences Unit, Cambridge, UK; University of Cambridge, Department of Experimental Psychology, Cambridge, UK; Medical Research Council Cognition and Brain Sciences Unit, Cambridge, UK

**Keywords:** Moral cognition, Mental models, Efficiency, Automaticity, Cognitive effort

## Abstract

A long-standing theoretical debate concerns the involvement of principled reasoning versus relatively automatic intuitive-emotional processing in moral cognition. To address this, we investigated whether the mental models formed during story comprehension contain a moral dimension and whether this process is affected by cognitive load. A total of 72 participants read stories about fictional characters in a range of moral situations, such as a husband being tempted to commit adultery. Each story concluded with a “moral” or “immoral” target sentence. Consistent with a framework of efficient extraction of moral information, participants took significantly longer to read immoral than moral target sentences. Moreover, the magnitude of this effect was not compromised by cognitive load. Our findings provide evidence of efficient coding of moral dimensions during narrative comprehension and demonstrate that this process does not require cognitively intense forms of principled reasoning.

Human moral cognition is currently a significant focus of neuroscientific inquiry (Greene & Haidt, 2002; Moll, Zahn, de Oliveira-Souza, Krueger, & Grafman, 2005), yet little remains known about the emotional and cognitive processes involved. In particular, one important yet outstanding issue is whether processing of moral information is a relatively automatic process or whether it requires significant cognitive effort (Greene & Haidt, 2002).

The study of moral judgement in both psychology and philosophy has been largely dominated by rationalist models that emphasize an important role for conscious reasoning in the judgement process. These models maintain that individuals make decisions about what is right or wrong by applying explicit reasoning principles to the particular circumstances of a given situation. Examples of two prominent theories grounded firmly in this type of thought are Piaget's and Kohlberg's theories of moral judgement in child development. Also consistent with this class of model is the suggestion that theory-of-mind judgements can be used as input in the moral cognitive process (Knobe, 2005). For example, if we are wondering whether we should blame a person for his behaviour (a moral judgement), we might first want to know whether his behaviour was intentional (a theory-of-mind judgement).

Other models have questioned the assumption that formal reasoning gives rise to moral behaviour and action and have placed a greater emphasis on the role of intuition and emotion in moral judgement (Greene, 2001; Greene & Haidt, 2002; Haidt, 2001; Hauser, Cushman, Young, KangXing, & Mikhail, 2007). These “social intuitionists” assume that people often make moral judgements without weighing personal concerns or ethical values, such as fairness and the law, and that moral judgements result from quick and automatic evaluations, or intuitions, instead. Support for this class of model comes from studies of “moral dumb-founding”, in which people have strong moral reactions without any plausible, rational principle that explains their reaction. To take an example described by Haidt (2001), imagine a scenario in which a sister and brother mutually consent to “sleep together” without anyone else knowing, with no harm done to either. Many people have a very strong negative reaction to this scenario yet cannot explain why using principled moral reasoning. Such findings, amidst others, have led Haidt (2001) to define moral intuition as the sudden emergence of an affectively valenced (e.g., good/bad) moral judgement in consciousness, with no awareness of the process of weighing evidence to arrive at some conclusion. This type of interpretation is consistent with the broader belief in the field of social cognition that many behaviours and judgements are made automatically and without intention, effort, or awareness of process (Bargh & Chartrand, 1999). It is also consistent with earlier psycholinguistic work on memory for the pragmatic implications of sentences. In this research, individuals often recognize or recall the implications of sentences rather than their actual content (Johnson, Bransford, & Solomon, 1973). Thus, when presented with the sentences “John was trying to fix the birdhouse. He was pounding the nail when his father came out to watch him and help him do the work”, participants falsely recognize the original sentence as having explicitly stated that a hammer was used. Similar effects have been reported for a wide range of materials.

One way to distinguish and evaluate the opposing reasoning and intuition models of moral processing would be to define controlled circumstances under which a situation requiring a moral judgement is implied, but not explicitly stated, and to test how efficiently it is assimilated. This would indicate whether processing of moral information shares certain characteristics with other mental processes considered to be “automatic”. As noted above, the emerging view in social cognitive and linguistic research is that many behaviours and judgements are relatively automatic; moral judgements might also be particularly habitual and susceptible to this kind of automation. The inability of individuals to provide a sound explanation for a particular moral stance may suggest involvement of emotion in moral processing, but it does not provide strong evidence of relatively automatic or efficient processing of moral information.

We adapted a paradigm used previously to investigate whether or not readers represent characters' emotional states during story comprehension (Gernsbacher, Goldsmith, & Robertson, 1992, 1998). Research has demonstrated that when people read stories, they form rich cognitive representations of the events, people, and settings described within; these representations have been termed situational or mental models (Johnson-Laird, 1983). To determine whether the emotional states of fictional characters are also represented in readers' mental models, Gernsbacher and colleagues conducted experiments in which participants read stories that implied, though never explicitly stated, a character's emotional state. Each story concluded with a target sentence that contained explicit reference to an emotion that either matched or mismatched the implied emotional state. For example, when reading a story about a woman who had just made her weekly visit to a nursing home and had learned that one of its residents had died, a reader forming an “online” representation of the character's emotional state during story comprehension will have incorporated the sadness the woman should be feeling into their mental model. The reader would subsequently be expected to read a matching target sentence (“She couldn't remember when she'd felt this much sadness”) faster than a mismatching target sentence (“She couldn't remember when she'd felt this much joy”) because the first sentence would be more readily incorporated into the reader's mental model. Gernsbacher et al. (1992) obtained this effect and argued that the kind of rich situational models formed during comprehension do indeed include attributes of characters' emotional states. Analysis of reading times for narrative outcomes has also been employed in other contexts. For example, Rapp and Gerrig (2006) demonstrated that participants were slower to read outcomes that were inconsistent with prior story contexts and preferences.

In the present study, we applied a similar reading-time methodology to a range of moral situations, such as a husband being tempted by his secretary to commit adultery. Each story concluded with either a moral or an immoral target sentence. If readers interpret the stories within a “moral” schema or framework during comprehension, then moral outcomes should be encoded more readily than immoral outcomes. This would subsequently be reflected in faster reading times for final matched sentences that describe a moral relative to an immoral outcome.

Insofar as any observed tendency for readers to process moral information in this way might be considered “automatic”, it has been suggested that this term does not refer to a single process that can be studied using a particular method or paradigm. Bargh and Chartrand (2000) maintain that a number of distinct qualities fall within this broad umbrella category of information processing: (a) whether an individual has some awareness of the operation of the process under consideration (e.g., moral processing); (b) whether the process is efficient; (c) whether the process is unintentional; and (d) whether the process is under conscious control. In the present study, we focus primarily on the second of these qualities of automaticity, the efficiency of moral processing. A process that is relatively unaffected by a reduction in conscious attention or cognitive resources for its successful operation could be regarded as efficient. One reliable method that has been used as a measure of efficiency of processing has been to manipulate a task's attentional demands and subsequently to observe whether this manipulation affects performance of the primary task. We manipulated task demands by comparing performance with and without a memory load presented at the beginning of each story. Initially, 24 participants completed the task with no memory load and 24 with a low memory load. To address whether increasing the memory load further had a signifi-cant effect on performance, a higher load was used with an additional 24 participants. For brevity, all three conditions are presented in the same methods and results sections. If moral attributes of readers' mental models are encoded efficiently, the reading time (RT) difference for immoral relative to moral target sentences should not be affected by memory load. However, an overall increase in reading times with increasing memory load would verify that the load is having its desired effect.

## Method

### Participants

A total of 72 adults were recruited from the Cognition and Brain Sciences Unit Volunteer Panel and were paid for their participation. All participants reported English as their first language, and verbal IQ was estimated from performance on the National Adult Reading Test (NART; Nelson, 1982). The data from one participant in the high-load condition were excluded from analysis as this participant's reading-time data were abnormally long (i.e., more than 3 standard deviations above the average). Descriptive statistics for the remaining 71 participants are provided in Table 1. Participants in the no-load, low-load, and high-load conditions did not differ in terms of the proportion of female to male participants, or in terms of age, *F*(2, 68) = 0.77, *p* = .47, $\eta_{p}^{2}=.02$, or verbal IQ, *F*(2, 68) = 2.14, *p* = .13, $\eta_{p}^{2}=.06$.

**Table 1:** Mean sample characteristics of participants in the cognitive load absent and present conditions

	Sample characteristic				
	
	N	Age (years)	n *female:* n *male*	Verbal IQ	Reasonable sentence completions (%)
No load	24	21.2 (0.8)	11 : 12	109.0 (1.2)	92.4 (4.0)
Low load	24	20.4 (0.5)	12 : 12	110.1 (1.3)	93.8 (2.0)
High load	23	21.7 (0.8)	13 : 11	112.7 (1.4)	92.0 (4.6)

*Note:* Standard errors of the mean are in parentheses. Verbal IQ was estimated according to the National Adult Reading Test (NART; Nelson, 1982).

### Materials and procedure

In pilot work, 24 experimental stories were developed to reflect a diverse range of moral themes, as reflected in Shweder and colleagues' (Shweder, Murch, Mahapatra, & Park, 1997) distinction of morality into different ethics or moral codes. The 24 stories were organized into 12 pairs such that both stories in each pair could conclude sensibly with either of two final target sentences, generating a “moral” and an “immoral” ending for each. These story endings were achieved by altering one word (in the example in Table 2, “wrong” to “right”). Further, the main characters were of the same sex in each pair and had names with the same number of syllables. Each story-pair was carefully written so that the same target sentence (e.g., “Jessica/Valerie thought about the situation and decided it would be wrong for her to do it”) would conclude one story in the pair with the character acting in a moral way and the other with the character acting in an immoral way (see Table 2). Thus, a target sentence that included the word “wrong” could be either moral or immoral conceptually, depending upon the context of the rest of the story. In this way, the “morality” of the final target sentence (i.e., moral or immoral condition) was always contingent on the preceding text.

**Table 2:** Sample pair of experimental stories (with moral and immoral target sentences)

Experimental story pair	
A.	Jessica had just moved in with her new boyfriend, Ben, and his daughter. Jessica's new life was idyllic as she was living in a beautiful house and no longer had to work. One night she came back early from an evening out with her friends. She discovered Ben physically abusing his daughter. She mentioned the situation to a friend who urged her to report Ben to the police.
	*Moral target sentence*
	Jessica thought about the situation and decided it would be right for her to do it.
	*Immoral target sentence*
	Jessica thought about the situation and decided it would be wrong for her to do it.
B.	Valerie had been married to Daniel for over a year. Unfortunately due to his job, she saw very little of him during the week. Recently she had noticed how attractive Daniel's best friend was. The friend occasionally came to her house to use Daniel's computer. She had all the opportunity she needed to seduce him while Daniel was at work.
	*Moral target sentence*
	Valerie thought about the situation and decided it would be wrong for her to do it.
	*Immoral target sentence*
	Valerie thought about the situation and decided it would be right for her to do it.

Participants were randomly assigned to one of four different computer-presented scripts. Within each script, the same target sentence was used for both stories in a moral–immoral pair (with the exception of the character's name). To take the pair of stories presented in Table 2 as an example, if Computer Script 1 used “Jessica (or Valerie) thought about the situation and decided it would be wrong for her to do it” as the target sentence for both stories in the pair, Computer Script 2 used “Jessica (or Valerie) thought about the situation and decided it would be right for her to do it” as the target sentence for both stories in the pair. In addition to allowing comparison of RTs within participants for “moral” and “immoral” target sentences of identical lengths, reversing the morality of both sentences in the pair across scripts ensured that morality was not confounded with valence across participants. Scripts 3 and 4 were identical to Scripts 1 and 2, respectively, but with experimental and filler stories presented in the reverse order to control for any order effects.

All four scripts presented the 24 experimental stories in a pseudorandom order, interleaved with 24 filler (or moral-neutral) stories. The fillers were those used by Gernsbacher et al. (1992): These were originally written for readers of American English and so were modified for British readers where necessary. The filler stories were moral neutral.

Participants were tested individually in a session lasting approximately one and a half hours. Following a short practice session, participants were instructed to read the 48 stories at a natural reading rate, one sentence at a time. Participants pressed the spacebar to proceed from one sentence to the next, and RTs for each sentence were recorded. RTs for the final, target sentence constituted the dependent variable. Participants were told that at the end of a selection of stories, they would be prompted to supply a reasonable concluding sentence to verify that they were attending to and comprehending each story's content. The filler stories were used for this purpose. No mention of morality or moral processing was made to participants.

Each participant completed one of three experimental conditions: (a) no cognitive load, (b) low cognitive load, or (c) high cognitive load. In the “no-load” condition, participants were asked to read through the scripts as detailed above. In the “low-load” condition, participants were given a memory load in the form of a trigram, or string of three letters (e.g., SDV), at the beginning of each story. This cognitive load was present for the duration of the story and associated target sentence. Participants were prompted to write down the remembered trigram once they had finished reading the target sentences for a selection of stories, though they had no knowledge of the stories on which the prompts might occur. Trials in which the letters and order were recalled accurately were recorded as “correct”. For the high-load task a six-digit memory load (e.g., 372815) was used. This provided a more stringent test of processing efficiency, on the basis of previous studies that have made claims about automaticity of social cognitive processes using this same cognitive load (Bargh & Chartrand, 2000; Bargh & Tota, 1988; see also Lavie, 2005, for a discussion of its use in cognitive tasks). Moreover, in the “high-load” condition, a six-digit memory load was presented at the beginning of each story, and every target sentence ended with a prompt to write down the corresponding digit sequence. The digit task also had the added advantage over the trigram (low-load) task in that the to-be-remembered stimuli could not be transformed into pronounceable nonwords or recognizable acronyms, although every attempt had also been made to ensure that this was not the case for the low (trigram) load task.

## Results

In order to make claims about the efficiency of moral processing in particular, it was important to show that our secondary tasks (memory loads) were of sufficient difficulty to adversely influence participants' overall performance on the primary task (reading times). The most representative measure of this variable was each participant's average reading time (RT) across all story sentences. A one-way analysis of variance (ANOVA) conducted on each participant's average RT per sentence showed a significant main effect of cognitive load, *F*(2, 68) = 4.55, *p* = .014, $\eta_{p}^{2}=.12$, with increased overall RTs observed for increasing cognitive loads (no load = 2,586 ± 135; low load = 2,656 ± 122; high load = 3,345 ± 292). Further analysis demonstrated a significant linear trend across memory load, *t*(68) = 2.73, *p* = .004, one-tailed, with an overall increase in RTs from no load to three letters to six digits, and no quadratic effect, *t*(68) = 1.30, *p* = .2 (i.e., no significant deviation from the linear). To exclude the possibility that increased RTs reflected an interaction between cognitive load and morality/immorality of the final sentence alone, a one-way ANOVA was also computed for the average RTs of all sentences except the final target sentences; the main effect of cognitive load on this RT measure was again found to be significant, *F*(2, 68) = 4.75, *p* = .012, $\eta_{p}^{2}=.12$.

**Figure 1:**
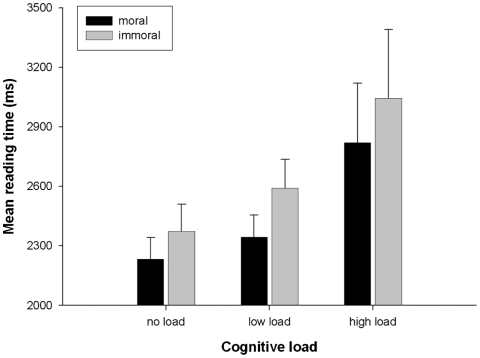
Mean reading times and standard errors of the mean for moral and immoral target sentences in the no-, low-, and high-cognitive-load conditions.

To examine RT differences for moral and immoral target sentences for each participant assigned to the no-load, low-load, and high-load conditions, mean RTs and standard errors were calculated separately for matched moral and immoral story endings; these mean RTs are presented in Figure 1. RT data were analysed using a 2 × 3 repeated measures ANOVA with story ending (moral, immoral) as a within-participants factor and cognitive load (no load, low load, high load) as a between-participants factor. The main effect of story ending was significant, with participants taking longer to read immoral than moral endings, *F*(1, 68) = 17.19, *p* < .001, $\eta_{p}^{2}=.20$ (see Figure 1). In line with the significant effect of cognitive load on overall RTs reported above, the main effect of cognitive load on RTs for final target sentences alone, *F*(2, 68) = 2.51, *p* = .08, $\eta_{p}^{2}=.07$, reflected a significant linear trend, *t*(68) = 2.17, *p* = .017 (one-tailed), with no significant deviation from the linear, *t*(68) < 1. Critically, the interaction between cognitive load and story ending was not significant, *F*(2, 68) < 1, $\eta_{p}^{2}=.012$. Thus, there was no evidence that slower RTs for immoral than for moral story endings were affected by the presence and size of the memory load.

For the low-load condition, 85.9 ± 13.1% of trigrams were recalled correctly upon prompting, whereas for the high-load condition, 80.2 ± 21.4% of digits were recalled correctly. These levels of free recall are comparable to those reported by Gernsbacher and colleagues (Gernsbacher et al., 1998) who employed both a four-consonant recognition-based memory load (88% accuracy) and a four-consonant cumulative memory load (81% accuracy) during narrative comprehension.

Across all participants, 92.7 ± 17.5% of completion sentences were found to be consistent with story content, indicating good comprehension; the percentage of acceptable completions did not vary significantly with cognitive load, *F* < 1, *p* > .5, $\eta_{p}^{2}=.002$ (see Table 1).

## Discussion

Our study provides new evidence that readers form mental models or representations that include information about the morality of fictional characters during narrative comprehension. We reasoned that if mental models of the thoughts and behaviours of fictional characters are interpreted and represented within a moral framework, increased RTs should be observed for target sentences that conflict with this framework. This hypothesis was confirmed: Participants took significantly longer to read final target sentences that described immoral than moral outcomes. Crucially, this effect was observed under conditions where both the length and valence of the target sentence were tightly controlled. This was achieved by pairing identical target sentences with different stories in order that the same sentence was interpreted as describing a moral or immoral outcome, depending on its preceding context. Increased RTs were also observed in the absence of any explicit instruction to attend to the moral implications of the particular events, behaviours, and thoughts described in the stories, suggesting that readers include this sort of information in their mental models without explicit intention. The present results accord with previous findings demonstrating that readers form rich representations that incorporate implied cognitive and emotional elements of narrative (Gernsbacher et al., 1992). Of more significance, they inform an important ongoing theoretical debate in the moral cognition literature.

Some theorists consider moral judgements to be made primarily on the basis of principled reasoning (Knobe, 2005; Kolhberg, 1969; Pizarro & Bloom, 2003), whereas others argue that quick and automatic evaluations, or intuitive-emotional processes, are critical in the judgement process (Greene, 2001; Greene & Haidt, 2002; Haidt, 2001). This was addressed by administering a concurrent low or high memory load to two thirds of our participants (three letters or six digits) for the duration of each story, including the final target sentence. Increasing cognitive load was found to increase reading time, as would be expected. Critically, however, there was no significant effect of load on the differential RTs for immoral and moral target sentences, the measure of the extent to which morally relevant information is integrated into the reader's mental model. Thus, finding that cognitive load does not interact with this immoral–moral differential indicates that the construction of a person's moral status is an efficient process that persists with an experimental reduction in cognitive resources.

It is important to emphasize that we are not claiming that the absence of an effect of cognitive load is specific to moral processing. Our experiment was designed to differentiate between two competing theoretical approaches to moral processing. One proposes that moral judgements are made on the basis of principled logical reasoning (Kolhberg, 1969; Pizarro & Bloom, 2003), the other that automatic intuitive-emotional processes are used (Greene, 2001; Greene & Haidt, 2002; Haidt, 2001). As discussed, our findings favour the latter. Moreover, consistent with the proposal that moral judgements often have an emotional basis, it is of interest that extraction of emotional information from emotion-based narratives is also resistant to similar cognitive loads (Gernsbacher et al., 1992). By contrast, cognitive load has been shown to influence the construction of mental models in other circumstances. For example, research into stereotype formation, in which participants read character descriptions followed by target sentences describing stereotype-consistent or stereotype-inconsistent behaviours, showed that reading times for the consistent and inconsistent target sentences interacted with cognitive load (Sherman, Lee, Bessenoff, & Frost, 1998).

Researchers in the area of social cognition have shown that many social psychological phenomena—including attitudes, evaluations and impressions, emotions, and social behaviour— occur automatically and without awareness (Bargh & Chartrand, 1999). The present findings suggest that the same may be true for moral processing. As emphasized in the introduction, automaticity should not be considered as a single process, but one that has multiple components (Bargh & Chartrand, 2000). We have provided evidence that at least one of these components applies to moral processing: It is an efficient process that persists despite a significant cognitive load. Given that our participants were not explicitly instructed to attend to moral aspects of the narrative content, our data indicate that another component of automaticity may also apply, that moral processing can under some circumstances occur without intention. Whether the processing of moral information shares further features of automaticity (e.g., whether it is under conscious control) is more difficult to assess and remains to be determined.

While the present data indicate that individuals process moral information in an efficient manner, they do not address the content of this processing. Allbritton and Gerrig (1991) have suggested that online processing may take the form of participatory or p-responses, a running commentary that occurs as the narrative unfolds, due to involvement in the text. To illustrate, in the example given above where a husband is tempted to commit adultery, the idea is that readers would hear an inner commentary that says “don't do it!”, and this is likely to have implications for reading times of the moral or immoral target sentence. A further interesting question is whether readers' mental models are based on their own personal moral or immoral attitudes and behaviours, or whether they are based on declarative knowledge of what is right and wrong. It is often the case that an individual who practises dubious morals may still possess adequate knowledge of what others consider to be right or wrong. This appears to be the case in the study of moral cognition in certain patient groups, such as psychopaths and patients with frontal lobe damage, where dissociations between explicit knowledge of how to behave and actual socially desirable behaviour have been observed (Anderson, Bechara, Damasio, Tranel, & Damasio, 1999; Damasio, 1994; Richell et al., 2003). Given that demand characteristics are frequently at play in the experimental setting, a major benefit of the type of paradigm adopted in the present study is that it provides a more subtle or implicit way of assessing the extent to which people assimilate moral knowledge and information. Moreover, the present approach could thus be useful in the study of moral cognition in these and other patient groups, which in turn may also contribute to our developing understanding of the neural basis of moral psychology.

In summary, these findings address the longstanding theoretical debate regarding the involvement of explicit reasoning versus intuitive-emotional processing in moral cognition. They provide support for the involvement of efficient, relatively automatic, and potentially intuitive processes in the construction of representations of implied moral dimensions in interpreting social scenarios.
